# Drug Delivery with Polymeric Nanocarriers—Cellular Uptake Mechanisms

**DOI:** 10.3390/ma13020366

**Published:** 2020-01-13

**Authors:** Levi Collin Nelemans, Leonid Gurevich

**Affiliations:** Department of Materials and Production, Aalborg University, 9220 Aalborg, Denmark; levinelemans@gmail.com

**Keywords:** drug delivery systems, endocytosis, polymeric micelles, amphiphilic block copolymers, nanoparticles, drug release

## Abstract

Nanocarrier-based systems hold a promise to become “Dr. Ehrlich’s Magic Bullet” capable of delivering drugs, proteins and genetic materials intact to a specific location in an organism down to subcellular level. The key question, however, how a nanocarrier is internalized by cells and how its intracellular trafficking and the fate in the cell can be controlled remains yet to be answered. In this review we survey drug delivery systems based on various polymeric nanocarriers, their uptake mechanisms, as well as the experimental techniques and common pathway inhibitors applied for internalization studies. While energy-dependent endocytosis is observed as the main uptake pathway, the integrity of a drug-loaded nanocarrier upon its internalization appears to be a seldomly addressed problem that can drastically affect the uptake kinetics and toxicity of the system in vitro and in vivo.

## 1. Introduction

**Figure 1:**
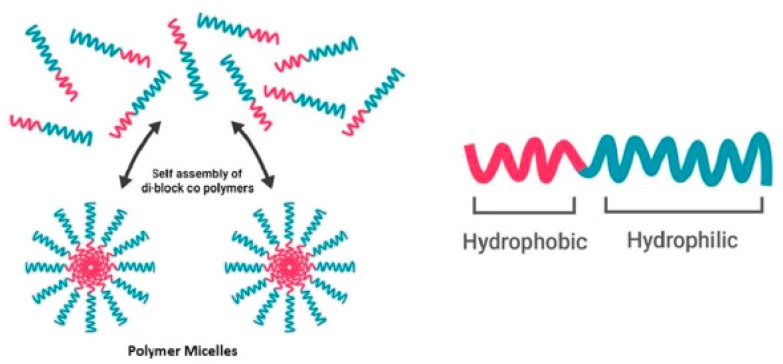
Above the critical aggregation concentration, amphiphilic block copolymers self-assemble into a micellular structure (adapted from Reference [8] with permission from Elsevier).

Nanocarriers have great potential as drug delivery systems (DDS). They enhance the bioavailability of drugs, extent circulation times and can accumulate in compromised tissue via an effect known as enhanced permeability and retention (EPR) [1,2,3]. In the past few years, the amount of nanocarriers in clinical trials has tripled [4]. Specifically, polymeric micelles have received growing attention due to their small size, simplicity and ability to transport hydrophobic drugs inside their core [3,5,6]. Furthermore, improved polymerization techniques lead to well-defined structures, narrow molecular weight distributions and tunable properties [1]. Polymeric micelles are composed of amphiphilic block copolymers (ABCs) containing hydrophobic and hydrophilic blocks [7]. When these ABCs are dissolved (in aqueous solution) at a concentration above their critical aggregation concentration (CAC), they will self-assemble into aggregates with a hydrophobic core and hydrophilic corona (Figure 1). Due to their low CAC and further stabilization due to a hydrophobic load, these polymeric micelles are relatively stable at working concentrations in the blood [1].

The hydrophobic block will influence the stability and drug release characteristics, while the corona influences the pharmacokinetic properties in vivo and can potentially be further modified (e.g., for active targeting, facilitated cell penetration and so forth. [1,9,10]). Poly(ethylene glycol/oxide) (PEG/PEO) is most commonly used as the hydrophilic block, while the hydrophobic block varies widely [11,12]. Poly(N-(2-hydroxypropyl)methacrylamide) (PHPMA) is a hydrophilic polymer that is a worthy competitor of PEG; it is biocompatible, non-toxic, non-charged and non-immunogenic [13]. Furthermore, the polymer contains hydroxyl moieties, which can be functionalized with targeting ligands, used for drug conjugation or facilitate other modifications that could potentially lead to the development of new micelle-based technologies [1]. Poly(N-vinyl-2-pyrrolidone) (PVP) has also been used to create polymeric micelles and potentially has the ability to cross membranes via biologically independent mechanisms, based on size [1,9,14]. It can be further modified with acrylic acid allowing a broad range of further modifications [15]. A relatively new class of polymer being used as DDS is poly(2-oxazoline) (POz). It is very versatile, and many different monomers can be produced with a wide variety of properties [1,16]. An example is poly(2-ethyl-2-oxazoline) (PEOz) which is used in polymeric micelles [17].

Polymeric micelles are intensively studied, and many excellent review articles give an overview of the composition of different polymeric micelles [2,7,18,19,20,21,22]. However, often only the cytotoxicity of these nanocarriers and their cargo are investigated, while the specific nanocarrier uptake and intercellular trafficking are mostly overlooked. The interaction between polymeric micelles and cell membranes is largely unknown and the fate of these polymeric micelles and their cargo after internalization remains to be clarified [6]. One of the concerns after internalization could be autophagy, which can greatly reduce the therapeutic effects of the drug [6,23]. Therefore, a better understanding of polymeric micelle uptake and drug release is crucial for creating an optimal DDS [24].

However, the uptake mechanisms of polymeric micelles are hard to generalize, since not only the physicochemical characteristics of the polymeric micelles but also the actual encapsulated drug and cell type play a crucial role in the uptake [25,26]. In this review, the uptake mechanisms for different polymeric nanocarriers are discussed, alongside with various experimental techniques commonly applied for discerning specific uptake mechanisms.

## 2. Endocytosis as the Main Uptake Mechanism in Cells

**Figure 2:**
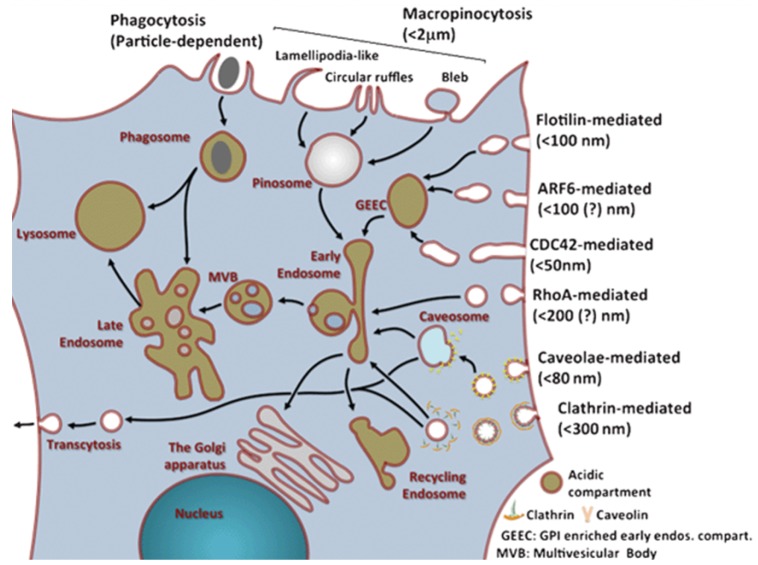
Overview of uptake and internal trafficking via various endocytic pathways in a typical eukaryotic cell, with an estimated maximum uptake size for different pinocytosis mechanisms (reproduced from Reference [24] with permission from The Royal Society of Chemistry).

Nutrients and other substances are mainly taken up by cells in a cellular process called endocytosis. Most nanocarriers are also thought to be taken up by this process. Traditionally, endocytosis can be divided into phagocytosis (uptake of large particles) and pinocytosis (uptake of fluids and solutes) (Figure 2). The latter can be further divided into macropinocytosis, clathrin-mediated endocytosis, caveolae-mediated endocytosis and clathrin- and caveolae-independent endocytosis. While these different uptake mechanisms have been extensively studied, they are still not fully understood. This is caused by the complexity of the processes, overlap of proteins involved in different mechanisms and the lack of mechanism-specific inhibitors [24].

### 2.1. Phagocytosis

**Figure 3:**
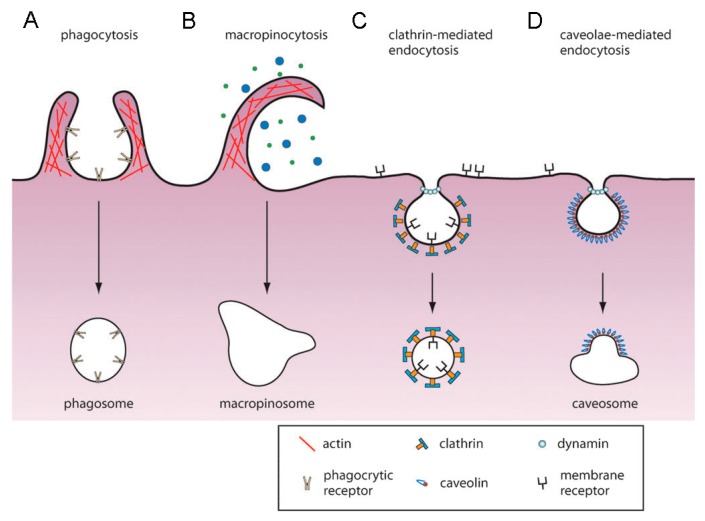
Uptake mechanisms of mammalian cells: (**A**) phagocytosis, **(B**) macropinocytosis, (**C**) clathrin-mediated endocytosis and (**D**) caveolae-mediated endocytosis. See text for more details. (Reproduced from Reference [32] with permission from The Royal Society of Chemistry).

**Figure 4:**
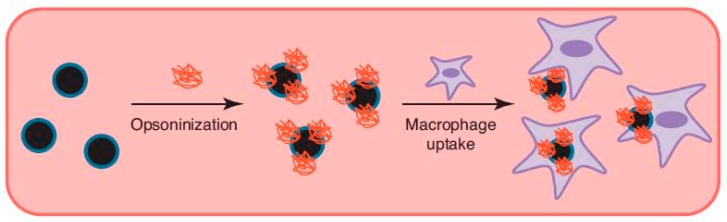
Opsonization of nanoparticles in the bloodstream will lead to rapid clearing by the reticuloendothelial system via phagocytic uptake of the particles by macrophages (reproduced from Reference [39] with permission from Elsevier).

Phagocytosis is only performed by a few specialized cells named phagocytes (macrophages, neutrophils, dendritic cells, etc.). Some other cells types, such as fibroblasts, epithelial and endothelial cells, might also display phagocytic behavior but to a much lesser extent [27,28]. The main task of phagocytes is to kill and remove pathogens, dead cells and cell debris. Phagocytosis is triggered via recognition of the particle via receptors on the phagocyte, which leads to recruitment of actin around the particle, followed by engulfment (Figure 3A) [29]. The created phagosomes are believed to eventually fuse with lysosomes, creating phagolysosomes. The acidic and enzyme-rich environment in these phagolysosomes will (promote) break down of any biodegradable nanocarriers or sensitive drugs [27]. Coating of the particle with immunoglobulins, complement proteins and other molecules enhancing phagocytosis (opsonins)—the so-called opsonization—will promote phagocytic uptake. Therefore, it is essential for a DDS to avoid opsonization, which will lead to subsequent clearing by the reticuloendothelial system (RES) from the bloodstream (Figure 4) [27].

The maximum size of nanocarriers that can be taken up via phagocytosis seems to be determined by the phagocyte’s cell volume. However, the particle shape might also influence the uptake. Champion et al. created polystyrene particles of different shapes and sizes and showed that the curvature of the particle at the initial point of contact determined the ability of the phagocyte to engulf the particle [30,31].

### 2.2. Pinocytosis

In contrast to phagocytosis, pinocytosis can be found in nearly all cells. Of the different pinocytosis mechanisms, *clathrin-mediated endocytosis (CME)* is the most studied to date and was first discovered in 1964 by Roth and Porter [24,33]. CME is mainly responsible for the uptake of essential nutrients, down regulation of cell signaling and maintaining cellular homeostasis (Figure 3C) [29]. In short, CME involves engulfment and upconcentration of transmembrane receptors bound to ligands on the plasma membrane. On the cytosolic side of the membrane, a coated pit is formed by cytosolic proteins, with clathrin as main unit [34]. These clathrin-coated pits are then pinched off the membrane by a small GTPase known as dynamin, forming clathrin-coated vesicles (CCV). Once the CCV is detached from the membrane, the coat will disassemble, and the vesicle will undergo further intracellular trafficking. Nanocarriers that enter the cell through CME are mostly targeted to degradative lysosomes. First, the cargo will be transported to early endosomes (pH ~ 6), which will mature into late endosomes (pH ~ 5). These late endosomes will fuse with prelysosomal vesicles to form lysosomes that have an acidic (pH ~ 4–5) and enzyme-rich environment (containing e.g., hydrolases) for degradation [27,35]. This pathway could be utilized to release the drug via biodegradation of the carriers only when the nanocarriers contain drugs that are stable under these harsh conditions. Otherwise, endosome escape strategies could be explored to optimize drug delivery [35,36,37].

*Caveolae-mediated endocytosis* (CvME) is another major uptake route responsible for biological functions, such as cell signaling, lipid regulation and vesicular transport (Figure 3D). The dimeric protein caveolin-1 (and caveolin-3 in muscle cells) is responsible for the specific flask shape of the vesicles and can be found as a striated coat on the cytosolic surface of the membrane [34]. As in CME, dynamin is responsible for scissoring of the vesicle from the membrane. These vesicles seem to fuse with caveosomes, thereby bypassing lysosomes. Therefore, CvME could be an interesting pathway for DDS to avoid lysosomal degradation [38].

*Macropinocytosis* is an endocytic process that entails engulfment of a large volume of the extra cellular milieu and is not directly driven by cargo (Figure 3B). This uptake is associated with membrane ruffling and can be induced by growth factors, bacteria, viruses and necrotic cells [24]. Some of these membrane protrusions can fall back onto the membrane and fuse with it, creating macropinosomes. These membrane protrusions are actin-driven and induced by the Rho-family GTPases [17]. Why only some protrusions result in micropinocytosis and how this process is regulated, is yet unknown. Macropinosomes are believed to fuse with lysosomal compartments, leading to degradation of the contents [27].

Cells that are depleted of CME and CvME still show some form of endocytosis. All these different uptake mechanisms are grouped together as *clathrin- and caveolae-independent endocytosis*. The uptake seems to be cholesterol dependent and involve lipid raft sorting on the membrane, however most pathways are still poorly understood [29]. A noteworthy example is the uptake of interleukin-2 receptors (IL-2), which seems to be clathrin- and caveolae-independent [34].

### 2.3. Elucidating Endocytic Pathways of Nanocarriers

A common way to analyze the uptake mechanisms of nanocarriers is by using endocytic inhibitors. When inhibition of a certain pathway drastically lowers the uptake of a nanocarrier, it is assumed to be responsible for nanocarrier uptake. However, most inhibitors are not specific to one endocytic pathway and may induce other side effects [5]. Furthermore, by inhibiting one specific mechanism, a secondary uptake mechanism might compensate, while it may not have been originally active [40]. These limitations to endocytic inhibitors are often overlooked, therefore the use of multiple inhibitors is recommended to verify the results. Table 1 gives an overview of some of the most used inhibitors with their main mechanism(s) and limitations.

Another, more precise approach for elucidating a specific uptake mechanism is the use of siRNAs. siRNAs can be used to reversibly inhibit the production of certain key proteins in endocytosis (e.g., clathrin, caveolin), which should reduce off-target effects [40,41,42]. Furthermore, it gives a better understanding of the involvement of certain proteins in endocytic pathways [43].

## 3. Uptake Mechanisms of Polymeric Micelles

The endocytic pathways for several classes of nanoparticles have been summarized in References [25,27]. However, despite the amount of research on polymeric micelles, data regarding the uptake mechanisms and intracellular trafficking of these micelles remains behind. Furthermore, most research that investigated the endocytic uptake of polymeric micelles only analyzed the uptake of the drug, hence, only providing indirect evidence for nanocarrier uptake [47,93,94,95,96,97,98,99,100,101]. As will be discussed below, this assumption may not always be correct, as the carrier and the load can separate upon the uptake. To proof nanoparticle uptake, the polymer should be labelled and colocalized with the drug. Labelling of the hydrophobic polymer segment is preferable, since changes in the charge of the corona can severely alter the uptake mechanisms of the polymeric micelles compared to unlabeled polymer [49,102,103,104]. Table 2 gives an overview of the uptake mechanisms of different polymeric micelles in mammalian cells. A better understanding of the fate of polymeric micelles and cargo after cellular uptake might prove useful in optimizing the efficiency of the DDS. Furthermore, when the fate and intracellular trafficking of the polymeric micelles is known, various release or escape strategies could be implemented for optimal drug delivery into the cytosol. These topics have been extensively reviewed by Varkouhi et al. [105] or with a specific focus on nanoparticles by Smith et al. [36].

### 3.1. PEO-b-PCL Micelles

Poly(ethylene oxide)-b-poly(ε-caprolactone) (PEO_44u_-b-PCL_20u_) micelles loaded with the fluorphore DiI were originally reported to be taken up via endocytosis, since the uptake was time, temperature, pH and energy dependent [110]. Uptake of conjugated rhodamine-PEO_45u_-b-PCL_23u_ micelles in P19 cells was also shown to follow an endocytic pathway, which provided direct evidence of uptake of the whole carrier [109]. Furthermore, conjugated TMRCA-PCL_23u_-b-PEO_45u_ micelles showed an increased uptake compared to the free model drug [3].

Kerdous et al. [111] has shown that pheophorbide-a (Pheo) loaded, PEO_5000_-b-PCL_4000_ micelles did promote cellular uptake in MCF-7 cells but did not alter the subcellular distribution of Pheo when compared to the free drug. To follow the kinetics, the fluorescent signal was measured over time. This showed that the uptake of the Pheo-loaded micelles involved two processes (a fast, high intensity and slow, low intensity), while the uptake of the free drug could be described by a single rate. Förster resonance energy transfer (FRET) analysis, by incorporation of both DiI (acceptor) and DiO (donor) inside the micelles, showed that the nanocarriers as such were not effectively taken up by the cells (4+ h), suggesting separation of the load (Pheo) followed by its rapid uptake (fast process) while uptake of the nanocarriers corresponded to the slow process. The observed effect was not caused by disassembly of the micelles in media, since the nanocarriers were found to be stable in culture media, within cell cultures and in the presence of proteins.

**Figure 5:**
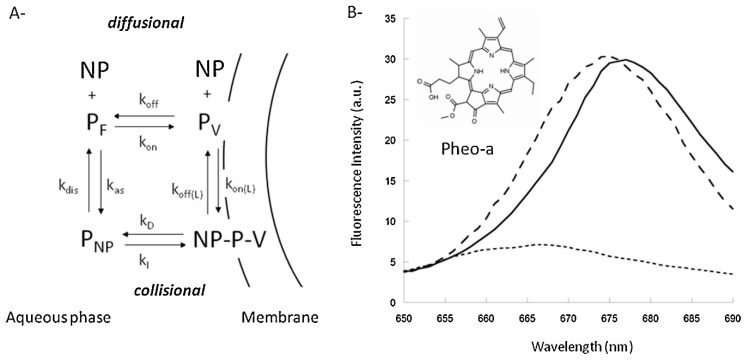
(**A**) Uptake of Pheophorbide-a (Pheo) loaded, PEO-b-PCL micelles might follow the collisional or diffusional kinetic mechanism. Which describes the free aqueous concentration of nanoparticles (NP), Pheo (PF), vesicles (V); Pheo associated to nanoparticles (PNP), vesicles (PV) and the Pheo-Nanoparticle-Vesicle complex (NP-P-V). (**B**) Pheo shows a different emission spectrum when present in DOPC vesicles (solid), nanoparticles (dashed) or phosphate buffered salin (PBS) (dotted), which can be used to investigate the uptake mechanisms of PEO-b-PCL micelles (reprinted from Reference [111] with permission from Elsevier).

Therefore, the uptake of the drug might be direct, via Brownian collisions between the nanocarrier and membranes (collision mechanism) or via drug diffusion through the aqueous phase (diffusion mechanism) before incorporation into the cell membrane (Figure 5A). These two proposed mechanisms are based on the theoretical model developed by Kuzelova et al. [112]. Large Unilamellar vesicles and the fluorescent sensitivity of Pheo to different environments (Figure 5B) were utilized by Kerdous et al. [111] to experimentally determine the uptake mechanism. Based on these kinetic models and experiments, it was suggested that the uptake of Pheo agrees most with the collision mechanism, in which there is direct transfer of the drug to the cell membrane.

Till et al. [108] investigated Pheo-loaded, PEO_2000_-b-PCL_2600/2800_ and PEO_5000_-b-PCL_4000_ micelle uptake in HCT-116 human colon cells and observed a similar effect as described by Kerdous et al. [111]. They suggested that the direct drug transfer might be facilitated by the PEO corona inducing dehydration of the lipid bilayer and enhancing membrane permeability [49,111,113].

Interestingly, when the nanocarriers were loaded with the fluorphore DiI, instead of Pheo, direct transfer was not observed [111]. Slow uptake of DiI-loaded nanocarriers was also previously shown by Maysinger (PEO_44u_-b-PCL_21u_) [114] and Mahmud et al. [101] (PEO_2000/5000/13000_-b-PCL_5000_ and PEO_5000_-b-PCL_13000/24000_). Pheo is less hydrophobic then DiI and might escape the nanocarrier more easily. These results show/suggest that not only the nanocarrier itself but also the drug can influence the uptake.

Therefore, Kerdous [111] and Till et al. [108] proposed that PEO-b-PCL micelles may be taken up differently depending on size and cargo. (1) Slow uptake, due to low penetration of the drug and carrier (e.g., DiI loaded), (2) drug release from carrier followed by transfer to the cell membrane (diffusion mechanism), (3) direct transfer (collision mechanism, Pheo loaded) of the drug between carrier and cell membrane.

These findings highlight the importance of not only tracing the drug but also the polymer in uptake studies. Several methods for labelling of polymeric micelles and their stability in various media were further reviewed by Savic et al. [115].

### 3.2. PEG-b-PLA

Monodisperse poly(ethylene glycol)-b-poly(lactide) (PEG-b-PLA) micelles were already produced by Yasugi et al. [116] in 1999 with a PDI of <0.1 and are still being investigated as a potential DDS [117].

**Figure 6:**
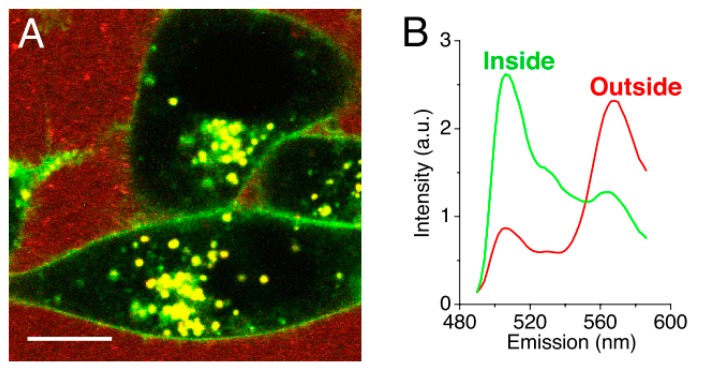
(**A**) Confocal fluorescence image of KB cells incubated with DiO/DiI loaded micelles. The image shows the loss of Förster resonance energy transfer on the cell surface and intracellular space. (**B**) Normalized spectra of the measured fluorescent signal outside (red) and inside (green) the cells. (Scale bar: 10 µm.) (Adapted from Reference [49], Copyright 2008 National Academy of Sciences).

Chen et al. [49] investigated the uptake of DiI loaded, fluorescein labelled PEG_5000_-b-PDLLA_5000_ polymeric micelles in KB cells. After a 24 h incubation, almost no uptake of the nanocarriers was observed, while the model drug was. The fluorphores DiI (acceptor) and DiO (donor) were used as a FRET pair to monitor the drug release into model membranes in real-time. The loss of FRET would be proportional to the uptake of the drug, since FRET only occurs when the fluorphores are in very close proximity to each other (below ca. 5 nm, loaded inside the nanocarriers). The results showed that the model membrane acted as a ‘sink,’ facilitating an efficient transfer between the hydrophobic drug and the membrane within minutes. The same uptake was observed in mammalian KB cells (Figure 6). Once inside the cell membrane, Chen et al. [49] suggested that the drug was further internalized via endocytosis, since both sodium azide and cytochalasin D treatment inhibited further uptake. This type of direct transfer seems similar to the one observed for PEO-b-PCL micelles described above.

**Figure 7:**
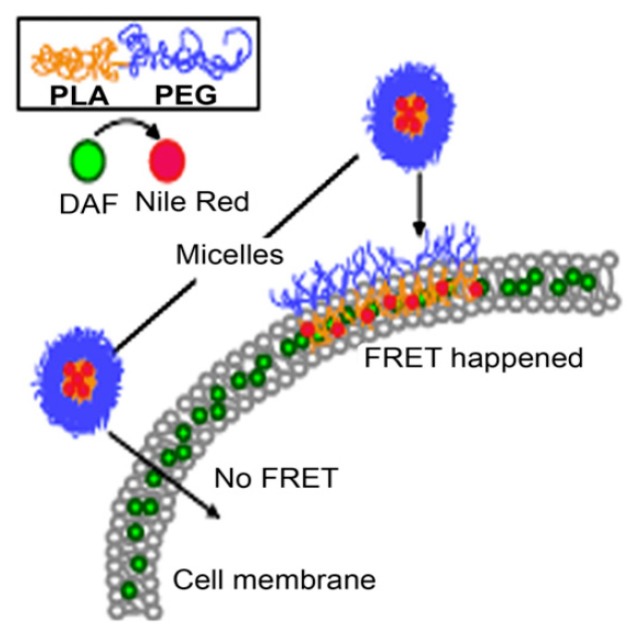
Förster resonance energy transfer will only occur if the micelles release their core loaded drug (Nile red, acceptor) into the DAF (donor) labelled cell membrane (Adapted from Reference [107] with permission from Elsevier).

This drug transfer to the cell membrane was also observed by Xiao et al. [107] in the ovarian cell line A2780. PEG_5000_-b-PLA_5000_ micelles loaded with the fluorphore Nile red (acceptor) and labelled cell membranes with DAF (donor), were used for FRET analysis. No FRET should be observed, if the whole nanocarrier would be taken up, since the distance between the donor and acceptor would be to large (Figure 7). However, Xiao et al. [107] showed that Nile red gets effectively and quickly released into the cell membrane (within 15 min). Although fusion of polymeric micelles with the cell membrane (as shown in Figure 7) cannot be directly proven with this method, supporting AFM data suggests roughening of the cell membrane upon exposure to micelles [107]. Nile red released into the membrane was further internalized using an endocytic pathway, in which lipid raft/caveolae-meditated endocytosis played a major role.

A follow-up study further investigated this specific uptake mechanism using wild-type or dominant negative forms of proteins, since chemical inhibitors can lead to nonspecific disruption in the cell [5]. The inhibitor dynasore indicated that the uptake was dynamin dependent, which was further confirmed with a dynamin-2 negative protein. The uptake was greatly reduced when using a negative caveolin-1 protein but clathrin did not seem to be involved in the uptake. In conclusion, uptake was deemed dynamin- and caveolin-dependent but clathrin-independent, in line with [107].

The uptake of PEG_3000_-PLA_40000_ nanoparticles was also investigated in Caco-2 cells, however only the uptake of the drug was measured [100]. Since drug-nanocarrier separation cannot be therefore excluded, the uptake did appear to be energy-dependent, lipid raft-mediated but caveolae-independent.

While such a direct drug release into a membrane might seem beneficiary in vitro, premature release of a drug to other hydrophobic compartments in vivo could greatly reduce the DDS’s efficiency. Cheng et al. [118] showed that intravenous (iv) injection of PEG-PLA micelles led to drug release and carrier breakdown within 15 min. This breakdown was mainly caused by association of the nanocarriers with alpha and beta globulins. Sun et al. [119] also showed that 80% of PEG-PCL/PLA micelles, upon iv injection, quickly dissociated into unimers. This effect was most likely caused by shear force and association with bloodborne proteins (particularly albumin). Therefore, the pharmacological effects of simple polymeric micelles might be limited in vivo.

### 3.3. PEG-b-PLGA

Poly(ethylene glycol)-b-poly(lactide-co-glycolide) (PEG-b-PGLA) seems to be a promising DDS [120,121,122], capable of passing the blood brain barrier [123,124]. Liu et al. [125] investigated PEG_2000_/_5000_-b-PLGA (different ratio’s) micelles on their biocompatibility and indicated that all micelles presented very low cytotoxicity. PEG_2000_-PLGA_7600_ micelles have been conjugated successfully to doxorubicin and the micelles showed a slow, steady release of the drug (over several weeks) and enhanced uptake, compared to free doxorubicin [126]. However, literature on the specific uptake mechanisms of this DDS is rather limited.

Zhang et al. [6] observed internalization of Courmarin-6 loaded, PEG-b-PLGA (Mn 10000) micelles in MCF-7 cells after treatment of two hours. The model drug was only located in late endosomes and lysosomes, possibly indicating that the model drug bypassed early endosomes. Since this effect is also observed in caveolae-mediated endocytosis, Zhang et al. [6] suggested this as the main uptake pathway. Since only the fluorescence of the drug was measured, no conclusions about the fate of the nanocarrier can be made. The uptake of the drug/nanocarriers further induced autophagocytosis, which the same group also observed with PEG-b-PLGA nanoparticles [23].

PEG_2000_-b-PLGA_5000_ micelles were previously created by Hu et al. [98] and the uptake in Calu-3 or NCI-H441 cells investigated [99]. After an incubation of one hour, the drug was observed in the cytosol. The uptake was deemed to be energy, cholesterol and clathrin dependent. Again, only the fluorescence of the drug was observed, which does not clearly indicate the fate of the polymeric micelles. However, the nanocarrier was loaded with both Nile red and Curcumin acetate and these could be colocalized.

## 4. Conclusions

Uptake of polymeric nanocarriers in mammalian cells is a complex process with many unknowns. Most of the reviewed studies indicate energy-dependent endocytic uptake, which can follow caveolae-, clathrin- or lipid-raft mediated pathways, as the main mechanism of internalization. As these pathways are interrelated and can be up or down regulated by a cell upon exposure to inhibitors, singling out a specific pathway is generally not possible. Interestingly, in many experiments where the loaded drug and the ABCs forming a nanocarrier were traced separately, drug-nanocarrier separation and direct drug transfer to the cell membrane were observed. While for in vitro experiments this additional mechanism can be considered as advantageous, greatly accelerating the uptake, it should be considered as indication of nanocarrier instability. Such a destabilization of nanocarriers can be particularly important in vivo, leading to opsonization, reduced circulation time, undesired drug distribution and toxicity due to incorporation of amphiphilic polymers into cell membranes. While some observations suggest that amphiphilicity or moderate hydrophobicity of the loaded drug can facilitate drug-nanocarrier separation, the factors leading to it have not yet received systematic experimental attention. Better understanding of this phenomenon and uptake mechanisms in general will lead to improved DDS with enhanced pharmaceutical efficiency and bring us one step closer to controlling the nanocarrier internalization mechanisms by design.

## Figures and Tables

**Table 1:** Overview of commonly used endocytic inhibitors, their effects and limitations [40,44,45,46].

Agent	Mechanism Affected ^1^	Effect	Limitation	Ref.
Low temp (4 degrees)	All energy dependent processes	Slows down/inhibits all energy dependent processes	Low temperature may influence fluidity of cell membrane	[47,48]
Sodium azide	All energy dependent processes	Inhibits respiratory system of cells	Toxic at higher concentrations	[49,50]
Chlorpromazine	CME	Translocates clathrin and AP2 from the cell surface to intracellular endosomes	Not efficient in all cell lines, might interfere with the biogenesis of intracellular vesicles	[51,52,53] [54] (pp. 19–20)
Cytosol acidification	CME	Inhibits the budding-off of clathrin- coated pits from the membrane	Interferes with macropinocytosis and the actin cytoskeleton	[54] (p. 19)
Hypertonic sucrose	CME	Removes plasma membrane-associated clathrin lattices	Nonspecific, interferes with fluid phase macropinocytosis	[54] (pp. 17–18) [55,56]
Monodansylcadaverine	CME	Stabilizes clathrin-coated pits	Induces global changes in actin dynamics	[54] (p. 20)
Phenylarsine oxide	CME	Mechanisms unknown, possibly a tyrosine phosphate inhibitor	Also inhibits micropinocytosis and is toxic at higher concentrations	[57,58]
Potassium depletion	CME	Removes plasma membrane-associated clathrin lattices	Nonspecific; affects actin cytoskeleton	[54] (p. 18) [59]
Dynasore	CME, CvME	Inhibitor of dynamin (small GTPase)	Has other off-target effects, including inhibition of membrane ruffling	[60,61]
Genistein	CvME	Inhibitor of several tyrosine kinases, causes disruption of the actin network	Affects several uptake processes	[62,63]
Okadaic acid	CvME	Phosphatase inhibitor, stimulates trafficking and internalization of caveolae	Nonspecific, off-target effects	[64]
**Cholesterol inhibitors**				
Filipin	CvME, Lipid raft	Binds to cholesterol in the membrane	Unstable and toxic, cholesterol influences other endocytic pathways besides CvME	[54] (pp. 23–24) [65,66]
Statins	CvME, Lipid raft	Lowering of cholesterol formation by inhibiting the enzyme 3-hydroxy-3-methylglutaryl CoA (HMG-CoA) reductase	Nonspecific, many off-target effects	[54] (p. 22) [67,68]
Methyl-β-cyclodextrin	CvME, Lipid raft	Removes cholesterol out of the plasma membrane by forming soluble inclusion complexes with cholesterol	Nonspecific, interferes with fluid phase endocytosis and CME, might induce membrane curvature	[54] (pp. 22–23) [69,70]
Nystatin	CvME, Lipid raft	Binds to cholesterol in the membrane	Toxic	[54] (pp. 23–24)
**(Endosome) acidification inhibitors**				
Monensin	Prevents acidification of endosomes	Acts as an ionophor, thereby inhibiting the acidification of endosomes		[71,72,73]
Nigericin	Prevents acidification of endosomes	Acts as an ionophor, thereby inhibiting the acidification of endosomes		[74,75]
Bafilomycin A1	Prevents acidification of endosomes	Inhibits the vacuolar ATPase endosomal proton pump.	Prevents maturation of autophagic vacuoles by inhibiting fusion between autophagosomes and lysosomes. Potentially inhibits Ca^2+^ pump SERCA	[76,77,78]
Chloroquine	Prevents acidification of endosomes	Increases pH of acidic vesicles (e.g., lysosomes), possibly inhibits some lysosomal hydrolases	Affects many other cellular processes	[79,80] (pp. 49–54)
Amiloride	Macropinocytosis	Inhibits macropinocytosis by lowering submembranous pH (cytosolic pH close to the membrane) and prevents Rac1 and Cdc42 signaling.		[81,82,83]
**F-actin inhibitors**				
Cytochalasin D	Macropinocytosis	Inhibits actin polymerization and may thus lead to actin filament disassembly	Nonspecific, may affect other endocytic processes	[54] (p. 26) [84,85]
Jasplakinolide	Macropinocytosis	Stabilizes actin and promotes actin assembly	Various effects depending on cell line and assay conditions	[84,86]
Latrunculin	Macropinocytosis	Sequesters actin monomers, blocks actin polymerization and may thus lead to actin filament disassembly	Not necessarily efficient in adherent cells	[54] (p. 26) [87,88]
Swinholide A	Macropinocytosis	Has F-actin severing activity	Nonspecific, may affect other endocytic processes	[86,89]
**Phosphoinositide 3-kinase inhibitors**				
LY294002	Macropinocytosis	Inhibits phosphatidylinositol 3-kinase class I and III	Nonspecific, also affects CME and CvME	[54] (pp. 26–27) [90,91]
Wortmannin	Macropinocytosis	Inhibits phosphatidylinositol 3-kinase class I and III	Nonspecific, also affects CME and CvME	[54] (pp. 26–27) [90,91]
3-methyladenine	Macropinocytosis	Inhibits phosphatidylinositol 3-kinase class III	Nonspecific, also affects CME and CvME	[54] (pp. 26–27) [92]

^1^ Abbreviations: Clathrin-mediated endocytosis (CME), Caveolae-mediated endocytosis (CvME).

**Table 2:** Overview of the proposed uptake mechanisms of different polymeric micelles.

Material ^1,2^	Uptake Mechanism(s) ^1^	Cell type ^3^	Drug ^1^	Comments	Ref.
Mixed micelles: TPGS2K, HS15, F127	Energy dependent CME CvME	Caco-2	Curcumin DOX	Only analyzed uptake of drug	[93]
OCC	CME CvME	Caco-2	Silybin Rhodamine-123	Only analyzed uptake of drug	[94,95]
OGC SH-OGC	CME CvME	Caco-2	Paclitaxel Rhodamine-123	Only analyzed uptake of drug	[96,97]
P(PEGMEMA)_75u_-b-PMMA_80u_	Clathrin and caveolae independent CME CvME	WiDr	DOX NileRed	80% of the uptake was via a different, undefined uptake mechanism	[106]
PEG_2000_-b-PLGA_5000_	Energy dependent CME	Calu-3 NCI-H441	NileRed Curcumin acetate	Only analyzed uptake of drug	[98,99]
PEG_3000/2000/5000_-PLA_40000_ PEG_2000/5000_-PLGA_40000_	Energy dependent Lipid raft mediated	Caco-2	Curcumin Coumarin 6	Only analyzed uptake of drug	[100]
PEG_5000_-b-PLA_5000_	Direct drug transfer to cell membrane Energy dependent Caveolae/lipid raft-mediated endocytosis	A2780	Paclitaxel NileRed FRET, DAF/NileRed		[5,107]
PEG-b-PLGA	CvME	MCF-7	DTX, 3-MA, CQ Coumarin 6	Only analyzed uptake of drug	[6]
PEO_2000/5000/13000_-b-PCL_5000_ PEO_5000_-b-PCL_13000/24000_	CME	MCF-7	DiIC	PEO5000-b-PCL13000 showed fastest uptake, only analyzed drug uptake	[101]
PEO_2000_-b-PCL_2600/2800_ PEO_5000_-b-PCL_4000_	Direct drug transfer to cell membrane	HCT-116	Pheo Conjugated Fluorescein		[108]
PEO_45u_-b-PCL_23u_	Energy dependent CME	P19	Conjugated Rhodamine		[3,109]
PEO_44u_-b-PCL_20u_	Temperature, pH and energy dependent	PC12	DiIC	Only analyzed uptake of drug	[110]
PEO_5000_-b-PCL_2000_ PEO_5000_-b-PDLLA_5000_	Direct drug transfer to cell membrane	KB	DiIC/DiOC Conjugated Fluorescein		[49]
PEO_5000_-b-PCL_4000_	Direct drug transfer to cell membrane	MCF-7	Pheo DiIC/DiOC	Micelle uptake is slow (>4 hr), while release of drug is fast	[111]
PEOz_6000_-b-PLA_1100/2200/3900/8500/10000/13700_ PEOz_2600/3300/4500/5600/6700/8900_-b-PLA_4000_	Energy dependent Cholesterol dependent Caveolae/lipid raft-mediated endocytosis	MCF-7	Paclitaxel Conjugated DEC	PEOz/PLA ratio of 1.7-2.0 for optimal uptake	[17]
Val-TPGS Phe-TPGS	Energy dependent CvME CME Macropinocytosis	Caco-2	Curcumin Coumarin 6	Enhanced transport across intestinal epithelial barrier, Only analyzed uptake of drug	[47]

^1^ Abbreviations: N-octyl-O, N-carboxymethyl chitosan (OCC), N-mercapto acetyl-N′-octyl-O, N″-glycol chitosan (OGC), poly(poly(ethylene glycol) methyl ether methacrylate)(P(PEGMEMA)), poly(methyl methacrylate) (PMMA), Poly(ethylene glycol)/poly(ethylene oxide) (PEG/PEO), poly(lactide-co-glycolide) (PLGA), poly(lactide) (PLA), poly(ε-caprolactone) (PCL), poly((D,L-lactide) (PDLLA), poly(2-ethyl-2-oxazoline) (PEOz), D-α-tocopheryl polyethylene glycol 1000 succinate (TPGS), Clathrin-mediated endocytosis (CME), Caveolae-mediated endocytosis (CvME), Doxorubicin (DOX), 5-dodecanoylaminofluorescein (DAF), Docetaxel (DTX), 3-methyladenine (3-MA), Chloroquine (CQ), 1,1′-Dioctadecyl-3,3,3′,3′-Tetramethylindocarbocyanine Perchlorate (DiIC), Pheophorbide-a (Pheo), 3,3′-Dihexyloxacarbocyanine Iodide (DiOC), 7-N,N-diethylamino-coumarin-3 (DEC). ^2^ When available, the average molecular weight of the polymer block is listed (Dalton). If the number of monomeric units was provided, it is denoted by a number followed by ‘u.’ If different sizes of the same polymer were used, they are listed separated by ‘/.’ ^3^ Information on the various cell lines: human colon cancer cell lines (Caco-2 and WiDr, HCT-116), human lung cancer cell lines (Calu-3 and NCI-H441), human ovarian cancer cell line (A2780), human breast cancer cell line (MCF-7), mouse pluripotent embryonic carcinoma cell line (P19), pheochromocytoma rat cell line (PC12), HeLa contaminant human tumor cell line (KB).
